# Development of a Noninvasive Real-Time Ion Energy Distribution Monitoring System Applicable to Collisional Plasma Sheath

**DOI:** 10.3390/s22166254

**Published:** 2022-08-20

**Authors:** Inho Seong, Sijun Kim, Youngseok Lee, Chulhee Cho, Jangjae Lee, Wonnyoung Jeong, Yebin You, Shinjae You

**Affiliations:** 1 Applied Physics Lab for PLasma Engineering (APPLE), Department of Physics, Chungnam National University, Daejeon 34134, Korea; 2 Samsung Electronics, Hwaseong-si 18448, Korea; 3 Institute of Quantum Systems (IQS), Chungnam National University, Daejeon 34134, Korea

**Keywords:** plasma, ion energy distribution (IED), real time, monitoring, noninvasive

## Abstract

As the importance of ion-assisted surface processing based on low-temperature plasma increases, the monitoring of ion energy impinging into wafer surfaces becomes important. Monitoring methods that are noninvasive, real-time, and comprise ion collision in the sheath have received much research attention. However, in spite of this fact, most research was performed in invasive, not real-time, and collisionless ion sheath conditions. In this paper, we develop a noninvasive real-time IED monitoring system based on an ion trajectory simulation where the Monte Carlo collision method and an electrical model are adopted to describe collisions in sheaths. We technically, theoretically, and experimentally investigate the IED measurement with the proposed method, and compared it with the result of IEDs measured via a quadrupole mass spectrometer under various conditions. The comparison results show that there was no major change in the IEDs as radio-frequency power increased or the IED gradually became broad as gas pressure increased, which was in a good agreement with the results of the mass spectrometer.

## 1. Introduction

Plasma processing in modern semiconductor manufacturing has attracted enormous interest in fabricating fine structures on wafers. For nanoscale electronic devices, conventional plasma technology is challenging to meet the demanded performance for the device. Therefore, advanced low-temperature plasma technology is required for high-level processes such as atomic-layer and high-aspect-ratio etching, which may satisfy zero-defect etching results and deep-and-narrow contact hole profiles [1,2,3,4,5,6,7]. To attain high-level processes, key process parameters such as etching and deposition rate, which determines the surface treatment results, were investigated to find their correlations to plasma internal parameters, namely, electron density, electron temperature, and ion energy [5,6,8,9,10,11,12]. Ion energy and its bombardment are important parts of physical etching such as sputtering and improving chemical surface reactions because they directly transfer energy to surfaces [13,14,15,16]. Ion energy thus needs be carefully optimized and controlled to meet the desired conditions. However, to monitor and precisely control ion energy distributions (IEDs) is very difficult; thus, this leads to the process drift or abnormalities causing low-productivity fabrication [17,18,19]. Therefore, a reliable method of IED control is needed for the next-generation process [20].

In academic areas such as universities, invasive diagnostics to measure the IED are retarding field energy analyzers (RFEAs) [21,22] and quadrupole mass spectrometer (QMSs) [23,24,25,26]. An RFEA measures ion fluxes overcoming grid potentials that repel ions of lower energy than that of their potential. IEDs are inferred by the first derivative of ion fluxes as a function of the grid potential [22]. A QMS comprises an energy analyzer, a quadrupole mass filter, and an ion detector, and measures ion fluxes with a specific energy that can pass through the analyzer. An IED is the measured ion fluxes by sweeping the potential of the energy analyzer [24]. To measure IEDs, such diagnostics are mounted onto an electrode surface or inserted into a reactor via a port. However, the semiconductor industry does not prefer to use such a diagnostic tool for the following several reasons. First, reactors used in the industry have few view ports because they can be a source of asymmetry in plasma uniformity. Second, an invasive method such as a QMS can perturb the plasma. To avoid this perturbation, a QMS can be placed onto the radio-frequency (RF) powered electrode, but a proper measurement is not allowed due to huge RF voltage interference to the QMS.

To overcome such issues from invasive diagnostics, an alternative is an ion energy monitoring system that measures and analyzes voltage waveforms on a powered electrode. Normally, voltage and current (V–I) probes used for arcing signal, particles, and etch end point detection are used by installing them behind a powered electrode [22,27,28,29,30,31,32,33,34]. The first attempt of the V–I probe as a diagnostic method is to estimate the plasma density and total ion current through simple analytical approaches from the measured electrode voltage and current waveform [34,35]. With continuous development, various plasma parameters, including an IED, can be obtained through a numerical sheath model that analyzes the measured voltage signal.

Sobolewski studied V–I waveforms between a powered electrode and impedance matching network, and developed models to estimate various plasma parameters with waveforms for a few decades [34,35,36,37,38]. He reported an ion flux and energy monitoring system with a collisionless sheath model [36,38]. Initially, the proposed technique was demonstrated with a bare metallic electrode without a wafer. Then, for practical use in plasma etching, the improved monitoring system with the high-speed data acquisition method was validated in a wafer-loaded environment in extremely low pressure [37]. However, the collisionless sheath model is not applicable for film deposition processing where the reactor pressure ranges several hundred mTorr. In this pressure range, the ion mean free path is shorter than the sheath thickness; as a result, the collision effect is included in the motion of ions in the sheath.

Recently, Bogdanova et al. have reported the feasibility of a virtual IED sensor based on the fast calculation of the ion trajectory simulation coupled to the Monte Carlo collision (MCC) method [39,40,41]. As the MCC increases the simulation time, to decrease the calculation time, the authors introduced a simple analytical model of a time-varying linear electric field and measured sheath voltage waveform that was used in the input data and defined as the subtraction of the powered-electrode voltage waveform from the plasma potential waveform. In this work, for rapid calculations, the plasma potential was implemented, and the following calculation time is investigated and optimized as a function of the number of injection particles. The sheath voltage waveform, which is a significant parameter to determine the shape of IEDs, is calculated as the difference between the potentials of the plasma and the powered electrode. Additionally, the simple analytical model of a time-varying linear electric field equation was used. Since actual plasma processing chambers do not allow for any invasive measurement, Bogdanova et al. investigated IEDs with a change in the plasma potential and the powered electrode voltage type: (i) invasive measurement of both voltage waveforms, (ii) assumption of plasma potential as sine, and (iii) assumption of both voltage waveforms as sine. Results show that, as assumptions are added, the accuracy decreases despite this being a noninvasive method.

In previous works, IED monitoring systems had the limitations of, for example, neglecting collisions, measuring invasively, and assuming the plasma potential to be a sine wave. To overcome these limitations, we propose a real-time IED monitoring system with an MCC model and an electrical sheath model. Instead of plasma potential measurement, the proposed system calculates it through the sheath model with a powered-electrode voltage waveform. To take into account collisions and give real-time feedback, we used the standard null collision method and simplified cross-sectional method for various collisions. We validated the resulting IEDs from our monitoring system by comparing them to the IEDs obtained with a commercial QMS in argon plasma at various conditions (RF powers, pressure). The result shows that the IED measured with the proposed monitoring system was in a good agreement with the QMS results, which shows the possibility of measuring accurate IEDs according to changes in various discharge conditions. The details of the methods and experimental setup of our IED monitoring system are described in the following section. The comparison results are shown and discussed.

## 2. Experiment

### 2.1. Vacuum and Plasma Conditions

**Figure 1:**
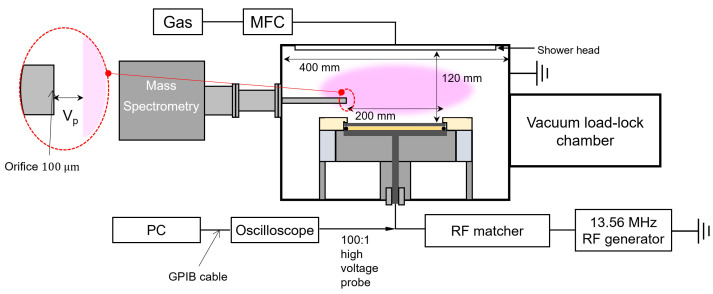
Schematic diagram of the experimental setup of the noninvasive IED monitoring system.

To validate the concept of the monitoring system, experiments were conducted with an asymmetric capacitively coupled plasma (CCP) source to which 13.56 MHz RF power was applied. Figure 1 shows a schematic of the CCP source where the diagnosis and determination of the plasma parameters such as a voltage waveform and IEDs were performed. The CCP chamber had a diameter of 200 mm and contained parallel-plate electrodes separated by 120 mm. The bottom electrode was surrounded by a ceramic ring for electrical insulation from the ground, and connected to a 13.56 MHz RF generator via an L-type matching network. The top electrode, which had a shower head to uniformly inject gases into the processing chamber, was grounded. A QMS (PSM, Hiden Analytic, Warrington, Cheshire, UK) that could measure IEDs by collecting energetic ions escaping from plasma was mounted to the chamber sidewall via a port. The vacuum system in this processing chamber consisted of a rotary pump and turbo molecular pump that arweree equipped to produce base pressure in the order of $10^{-6}$ Torr. We injected 100 sccm Ar gas through the shower head, and a variable-conductance valve between the processing chamber and the turbo molecular pump controlled the pressure.

Since various internal and external parameters, electrode voltage, and pressure can affect the profile of IEDs, the experiments were carried out by changing the applied RF power from 100 to 500 W, and the pressure from 10 to 50 mTorr to validate our noninvasive IED monitoring system. The ions were accelerated with a time-varying electric field in the sheath near the powered and grounded electrode, and the chamber wall. IEDs mostly strongly depend on the electric field in the sheath in several types of acceleration and collisions.

### 2.2. Diagnostics

IEDs were measured with a QMS mounted on the processing chamber sidewall for a comparative study [23,26]. This spectrometer comprised the inline energy analyzer *Bessel box*, a triple-filter quadrupole analyzer, and an ion counting detector with a differential pump housing composed of a rotary pump and turbo molecular pump that maintained the base pressure kn the order of $10^{-7}$ Torr for ions to transit into the QMS without collisions to the detector [24]. Ions accelerated in the sheath flowed into the chamber of the QMS through a 100 μm orifice located at the end of a grounded sampling probe, but since the commercial sampling probe that contained the orifice could not sample ions in this system, the sampling probe had to be extended and be exposed to plasma. Ions that flowed into the chamber of the QMS through the grounded sampling probe were focused by the electrical lens system to obtain high transmission before reaching the entrance hole of the energy analyzer that was composed of a cylindrical vessel and two circular plates connected to the different voltage sources. Sweeping the voltage sources that are fixing the potential distribution in the analyzer, IEDs could be measured. Ions resolved from the energy analyzer flowed into the quadrupole analyzer where four parallel rods equidistant from the central axis were biased with RF and direct current (DC) voltages, with the opposite rods being at the same potential. Since the RF and DC voltages determine which mass of ions would pass through the quadrupole analyzer, they were set to measure the energy distribution of argon ions in these experiments. Filtered ions need to be transformed from the current into readable signals, which is carried out in the ion detector.

### 2.3. Validation

To test the validity of our proposed concept, the QMS result and our result were compared by comparing both IEDs on the grounded electrode (or wall) rather than powered electrode, because the commercial QMS of which the sampling probe was grounded could only measure the IED on the grounded electrode. Our IED monitoring system, which can obtain IEDs on a both powered and grounded electrodes, needs a voltage waveform on a powered electrode as an input parameter provided by high-voltage probe measurement with oscilloscopes.

## 3. Noninvasive Monitoring System

**Figure 2:**
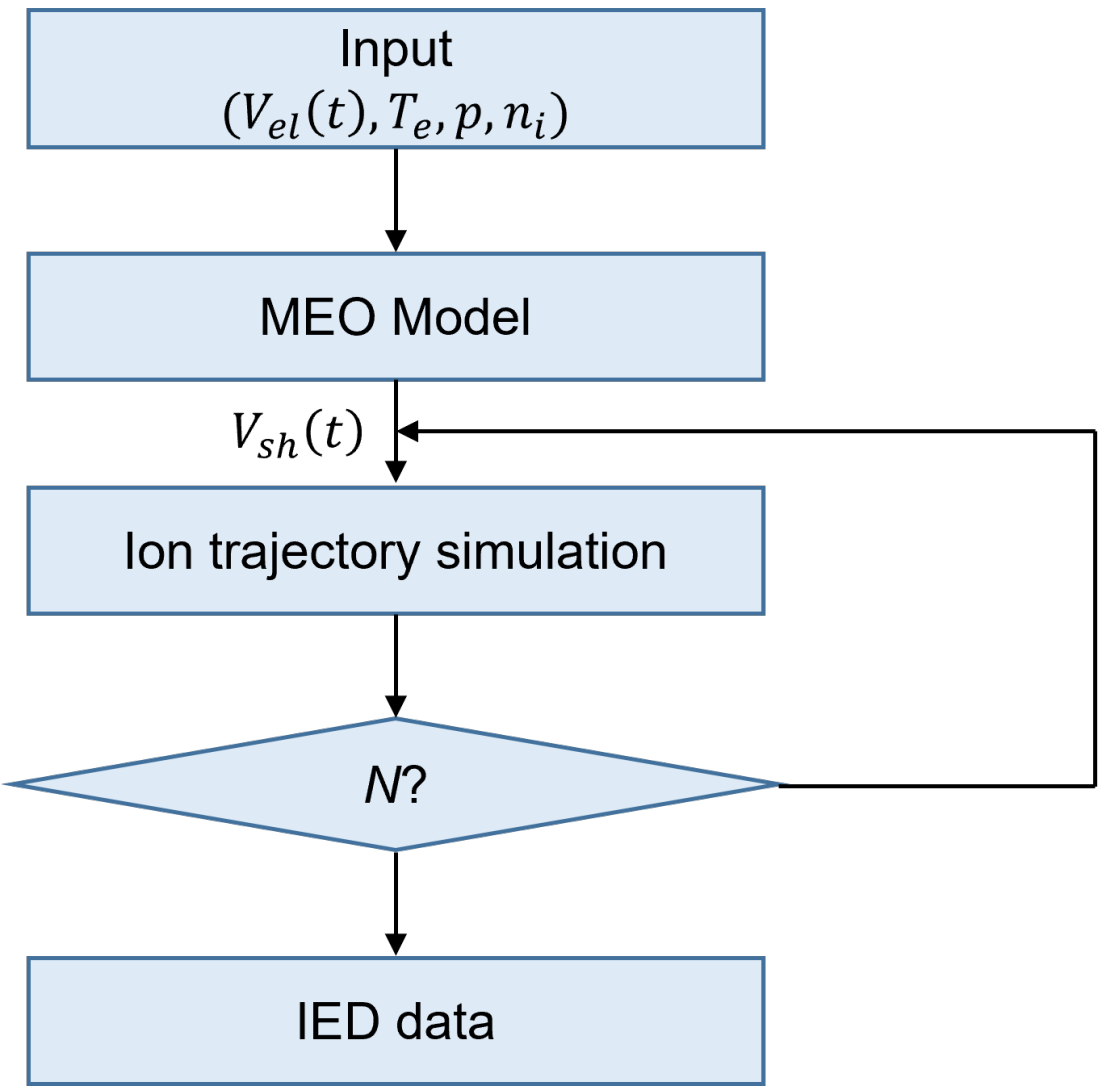
Flowchart of the simulation algorithm in the noninvasive IED monitoring system.

Since the concept of our IED monitoring system is noninvasive, it is combination of hardware for the measurements of electrode voltage as an input parameter and model-based software for analyzing voltage measured by the hardware and describing ion motion. This system was based on a flow chart for input-parameter acquisition, electrical-model calculation, ion-trajectory simulation, and IED data acquisition as shown in Figure 2. Since another concept of this system is real-time monitoring, various methods, such as simplifying the cross-section of ion-neutral collisions, were used for cost calculation. Details on the hardware, software, and speed-up are outlined later.

### 3.1. Input Parameter

Since IEDs strongly depend on the sheath voltage waveform, it is important to take exact waveforms of the sheath voltage, which can be obtained by subtracting the electrode voltage from the plasma potential. In previous research, the plasma potential was measured via an RF antenna inserted into plasma. Because this method is not applicable to our noninvasive one, the powered electrode voltage was measured instead of it [40].

The electrode voltage waveform was measured with a commercial high-voltage probe (P5100, Tektronix Inc., Beaverton, OR, USA) that was readily available, and mounted between the electrode and the matching network as shown in Figure 1. The waveform was digitized with an oscilloscope (TDS3054B, Tektronix Inc., Beaverton, OR, USA) that was linked to the computer with lab-produced software (MATLAB2018, Natick, MA, USA) via a general-purpose interface bus (GPIB) cable.

The analysis of electrode voltage allows for obtaining the plasma potential via electrical sheath model Metze, Erine, and Oskam (MEO) [42]. The details of this model are described in Section 3.2.1. Thus, this model was used to obtain the plasma potential without any invasive diagnostics in our system, which requires the electrode voltage waveform as an input parameter. The sheath voltage waveform was obtained by subtracting the calculated plasma potential from the measured electrode voltage.

### 3.2. Model

#### 3.2.1. Electrical Sheath Model

**Figure 3:**
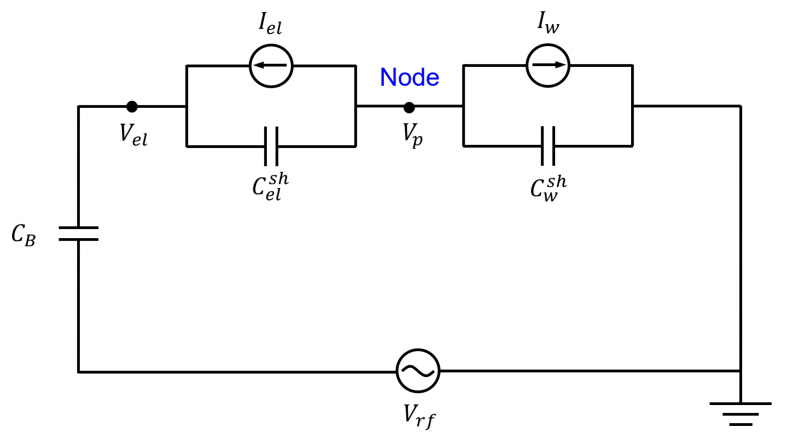
Schematic diagram of the equivalent circuit for the RF sheath model.

The model of Metze et al. was applicable to calculating the plasma potential in a fast feedback concept, although this model becomes more accurate as the applied field approaches the low-frequency regime where ions and electrons both respond instantaneously to an imposed time variation of the sheath voltage [42]. In this section, the MEO model adjusted in our system is explored. A schematic of the equivalent electric circuit model for the plasma reactor is shown in Figure 3. Here, $V_{rf}$ is the voltage of the applied RF signal from the matched RF generator, $V_{el}$ is the voltage applied on a powered electrode through a blocking capacitor of which capacitance is represented as $C_{B}$, and $V_{p}$ is the plasma potential. The sheath capacitance and conduction current adjacent to the powered electrode are represented as $C_{el}^{sh}$ and $I_{el}$, respectively, while the corresponding values adjacent to the grounded wall are $C_{w}^{sh}$ and $I_{w}$. $V_{el}^{sh}$ and $V_{w}^{sh}$ represent the voltages across the grounded wall-plasma sheath and the powered electrode-plasma sheath, respectively, but those are not shown in Figure 3. Since the details of the derivation and analytic expressions for the sheath capacitance and conduction current were clearly described in [42], they are omitted in this work.

The purpose of our system was to obtain a time-varying plasma potential from the powered electrode voltage measured via a voltage probe through this electric circuit. From this equivalent circuit and the current conservation law, as shown in Figure 3, it can be expressed as follows:

(1) $$C_{w}^{sh}\frac{\partial V_{p}}{\partial t}+I_{w}+C_{el}^{sh}\frac{\partial (V_{p}-V_{el})}{\partial t}+I_{el}=0.$$

**Figure 4:**
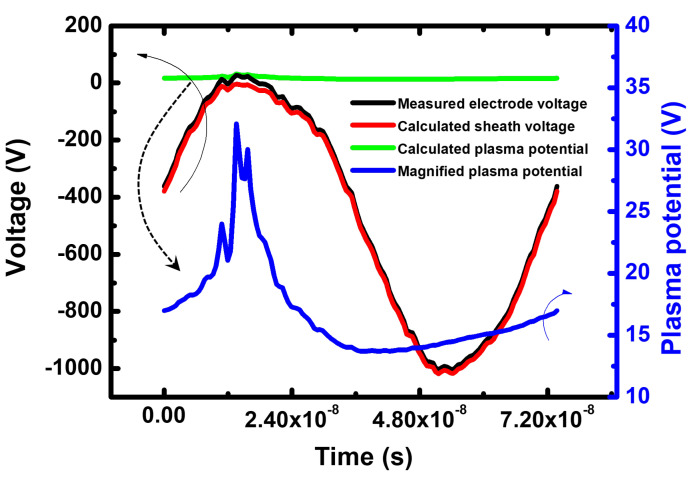
Measurements of electrode voltage, sheath voltage adjacent to the powered electrode, plasma potential, and magnified plasma potential.

If the analytic expressions for $C^{sh}$ and *I* of both sheaths from [42] are used in Equation (1), time-varying plasma potential $V_{p}$ can be numerically obtained. To solve this partial differential equation, the Runge–Kutta fourth-order method was used. Figure 4 shows the calculated waveforms for electrode voltage $V_{el}$, plasma potential $V_{p}$, sheath voltage $V_{el}^{sh}$ adjacent to the powered electrode, and magnified plasma potential in Ar plasma at a pressure of 20 mTorr and RF power of 300 W. The calculated plasma potential was similar except for the phase when the electrode voltage was positive. The sheath voltage always had negative potential with respect to the plasma, which was in a good agreement with measurements by Bruce [43].

Since plasma processing such as surface modification and etching is performed on a powered electrode, it is greatly meaningful to obtain and analyze the sheath voltage waveform adjacent to that electrode. Nevertheless, since the QMS can only measure IEDs at the grounded sheath due to the grounded sampling probe, we obtained the sheath voltage waveform adjacent to the grounded electrode for the IED comparison between our monitoring system and the commercial QMS, as mentioned in Section 2.3.

#### 3.2.2. Ion Trajectory Simulation

In order to obtain the IED on the grounded or powered electrode, the sheath was simulated on the basis of a simple matrix sheath model, with sheath voltage waveforms obtained from the measured voltage adjacent to the powered electrode with the MCC algorithm for ion-neutral collisions [44,45]. From the voltage adjacent to the grounded electrode, which was equal to the plasma potential, shown in Figure 4, an electron sheath edge s(t) was calculated as follows:

(2) $$s\left(t\right)=\sqrt{\frac{\epsilon_{0}V_{p}\left(t\right)}{2en_{0}}},$$

where $\epsilon_{0}$ is permittivity in vacuum, $V_{p}\left(t\right)$ is the time-varying plasma potential, *e* is an electron charge, and $n_{0}$ is a constant ion density. If the sheath adjacent to the powered electrode is considered, $V_{p}\left(t\right)$ should be replaced with the absolute value of the sheath voltage on the side of powered electrode, which is always negative with respect to the plasma. Spatially linear electric field *E*(*x,t*) in the sheath was derived from Poisson’s equation as below.

(3) $$E\left(x,t\right)=\left\{\begin{array}{cc} \frac{en_{0}}{\epsilon_{0}}\left(x-s\left(t\right)\right), & x<s(t)\\ 0, & x\geq s(t). \end{array}\right.$$

To solve the ion trajectory simulation, boundary conditions such as injected ion velocities into the sheath and the initial plasma phase for each ion at t=0 for consideration on one RF period should be defined. Here, ion incident velocities at the ion sheath edge where quasineutrality is always satisfied during discharge are given by

(4) $$u_{B}=\sqrt{\frac{eT_{e}}{m_{i}}},$$

where $T_{e}$ is the electron temperature, and $m_{i}$ is ion mass. This velocity is technically termed as Bohm velocity, and $u_{B}$ is a criterion to keep the quasineutrality of plasma [46]. Because the motion of ions can vary according to the plasma phase as they pass through the ion sheath edge, in this simulation, ions were launched from all phases in one RF period.

The model consisted of three coupled modules, namely, the field solver, particle mover, and MCC model. First, the electric field was calculated by the field solver for two cases: (i) if ions in the plasma phase, and the electric field being zero; and (ii) if ions in the sheath phase, and the electric field following the equation specified above. The second is the particle mover modulus in which the motion of the ions is determined by the force equation with the electric field calculated in the field solver. Third, in order to take collisions into account, we used the MCC method from which the probability of the elastic and charge exchange collisions is calculated. Electron impact collisions such as ionization and dissociation were assumed to be negligible in this monitoring system. In thermal equilibrium, the density of electrons satisfies the Boltzmann relation because electrons are highly mobile. Therefore, electron-neutral collisions can be neglected because the electron density was small in the sheath, of which the voltage was much higher than the electron temperature. The distance was calculated from the particle mover in the sheath during the time interval. A comparison between the probability for each collision and a random number between 0 and 1 determinded whether the electrons collide with neutral gas or not. These steps were repeated until the ion reached the electrode.

### 3.3. Speed Up

One of the key properties of our monitoring system is real-time measurement. The calculation time of a conventional IED monitoring system is a few minutes, however, which suggests that it is not likely that the industry would adopt the monitoring system in their processing. Therefore, the calculation time of our monitoring system had to be shorter to meet the industrial demand while the data quality should not deteriorate. We introduce the three methods to reduce the calculation time for one IED. The IED calculation times were investigated through a simple ion trajectory simulation where arbitrary sinusoidal sheath voltage waveforms were entered with amplitude of 300 V and pressure of 20 mTorr.

**Figure 5:**
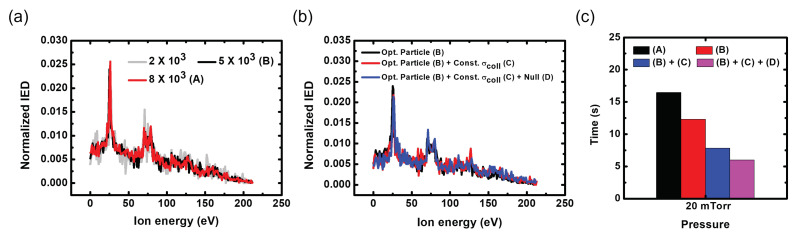
(**a**) Calculated IEDs according to the number of ions; (**b**) calculated IEDs; (**c**) corresponding calculation times with additional speed-up methods. (A) Nonoptimized particles 8 × $10^{3}$ without speed-up methods; (B) optimized particles of 5 × $10^{3}$ without speed-up methods; (C) constant cross-section method; (D) null collision method.

First, the number of calculated particles was optimized to minimize the calculation time without the loss of information on the IED profiles. As shown in Figure 5, in the case of an IED with 2 × $10^{3}$ particles, there was noise that blurred which signals were reasonable over the entire energy range. On the other hand, the noise was greatly reduced in the case of 5 × $10^{3}$ (B) and 8 × $10^{3}$ (A) particles, especially at the lowest energy and near 50 eV. Figure 5a shows that the calculation time of 8 × $10^{3}$ particles was longer than that of the 5 × $10^{3}$ particle case, even though the IED profiles were similar to each other, as seen in Figure 5c. Thus, we used the particle number of 5 × $10^{3}$ that guaranteed good IED quality and the shortest calculation time. Second, the cross-section of collisions was simplified to be constant. The function of fitting curves is well-known for the experimental cross-sectional data of argon ion-neutral collisions [47]. Conventionally, the fitting function of the cross-section was calculated as a function of the energy of the ions in the sheath.An investigation of the calculation time for each code provided by MATLAB confirmed that the cross-sectional calculation takes a long time. Therefore, the cross-section was simplified to a constant with the following steps. Before calculating one IED, we (1) acquired the maximal sheath voltage measured at the target electrode (powered or grounded), and (2) averaged the cross-section from zero to the maximal sheath voltage. The collision cross-sections obtained in the following steps were employed for all collisions in the ion trajectory simulation. Third, we used the well-known null collision method [48] calculates the number of ions involved in a collision using the maximal probability involved in a collision at single time step and randomly selects ions from among all ions. Figure 5b shows the calculated IEDs according to additional speed-up methods such as the constant cross-section of collisions (C) and null collision (D), including the optimization of calculated particles (B). The profiles of the IEDs according to the additional methods were almost the same, but the calculation time was shorter than 10 s, as shown in Figure 5c. However, since 10 s is also a long time to give fast feedback in current industrial process systems, we improved this simulation by using the vectorization of the code and loading a time-varying electron sheath edge from a database instead of calculation; we achieved a calculation time of a few seconds.

## 4. Results and Discussion

The accuracy of our noninvasive IED monitoring system was determined by how the system could produce the IED profile and the IED measured via commercial QMS depending on the RF powers and pressures. The y-axis unit of the IED in the commercial system and our system was different before data processing. In the case of our system, the y axis was the number of ions for that energy; in the case of QMS, it was the number of ions counted per second. Therefore, since the integration of an IED is a parameter representing the total number of ions, we normalized the IED to the integration value of each IED for the comparison.

**Figure 6:**
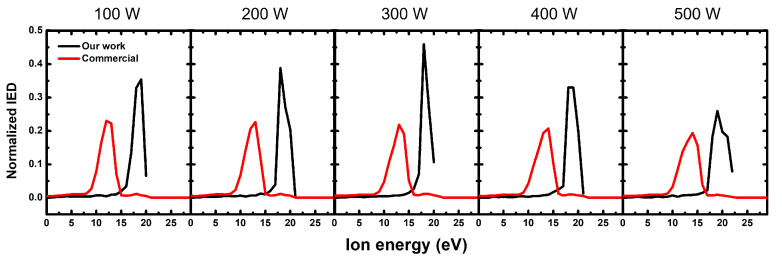
Normalized IEDs measured via our noninvasive monitoring system and by a commercial QMS with an increase in RF power from 100 to 500 W at a fixed pressure of 20 mTorr.

Figure 6 shows the comparison between the IEDs measured via our noninvasive monitoring system and the commercial QMS with increasing RF power at a fixed pressure of 20 mTorr in Ar plasma. Figure 6 shows that the measured IEDs via the commercial QMS had a single energy peak because, at the frequency of this input RF power higher than the ion frequency, the ions could respond to a time-averaged sheath potential that was equal to a time-averaged plasma potential at the grounded electrode. As the input RF power increased, the energy peak also slightly changed from 12 to 13 eV because it did not significantly affect the plasma potential, but RF and DC self-bias voltages applied on the powered electrode increased [49,50,51]. The IED calculated with our monitoring system showed similar changes according to the power variation measured via the commercial QMS, which implies that IEDs of both our and commercial diagnostics were in good agreement with each other.

**Figure 7:**
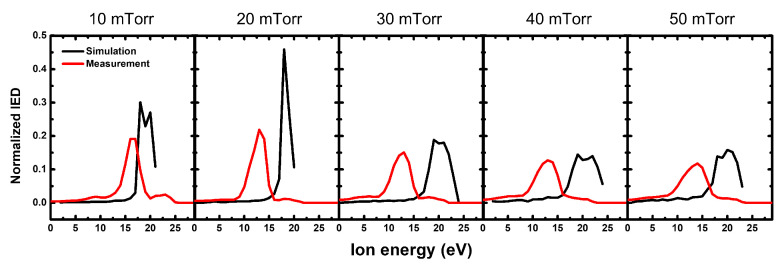
Normalized IEDs measured via our noninvasive monitoring system and by a commercial QMS with an increase in pressure from 10 to 50 mTorr at a fixed RF power of 300 W.

Figure 7 plots the IEDs of both diagnostics as a function of pressure ranging from 10 to 50 mTorr at a fixed input RF power of 300 W. The IEDs obtained from the commercial QMS with increasing pressure had similar trends of a single energy peak, and that changed with power variation. From the aspect of a single energy peak, there was a slight change because as chamber pressure changed from 10 to 50 mTorr, the electron temperature barely changed, which implied that the plasma potential remained almost unchanged. With increasing pressure, the ion energy distribution caused by the collision is broadened [45,52,53]. Figure 7 shows that the IED measured via the commercial QMS became broader as chamber pressure increased, which also appeared in the IED calculated with our monitoring system.

Nevertheless, while the amplitude of the peak in the IED measured with the commercial QMS did not change with the change in external conditions, the amplitude of our system tended to change, which may be attributed to two limitations of our system. First, the error of the voltage measurement at the powered electrode could have caused the difference between both spectra in Figure 6 and Figure 7. In the case of our monitoring system, the IED greatly depended on the sheath voltage at a ground electrode, which was obtained from the measured voltage applied to the powered electrode through the model. This obtained sheath voltage was small compared with the measured voltage. and was easily affected by the noise of the measured voltage. For this reason, the variation in their amplitude may have been caused as a function of RF input power due to the error of the measured voltage or the limitation of the measuring equipment. Second, since the electrical sheath model of our system was mainly used for low-frequency analysis, there was a limit to the application of the high-frequency regime. The displacement current, defined as $\frac{\partial }{\partial t}\left(C_{sh}\left(t\right)V_{sh}\left(t\right)\right)$, where $C_{sh}\left(t\right)$ and $V_{sh}\left(t\right)$ are time-varying sheath capacitance and voltage, respectively, created the harmonics of the sheath oscillation, which resulted in the plasma potential oscillation. The displacement current rarely influences the total discharge current in a low-frequency regime, and the plasma potential waveform becomes not sinusoidal but highly nonlinear, as depicted in Figure 4. This potential waveform resulted in a narrow IED, as most ions were accelerated in the flat potential region. On the other hand, since the displacement current dominates in a high-frequency regime, the plasma potential became sinusoidal. However, since the time-varying sheath capacitance is ignored in the MEO model, this high-frequency effect worked weakly on calculating the waveform of the plasma potential in our monitoring system. If a model is used that considers the time-varying sheath capacitance, the sheath voltage becomes a more sinusoidal waveform. In other words, the IED became broader than the IED provided by our monitoring system, and the amplitude of their peak decreases because ions were energized by sinusoidally oscillating waveform of the sheath voltage. An improvement in the electrical sheath model is our next challenge for high accuracy in high-frequency sheaths.

**Figure 8:**
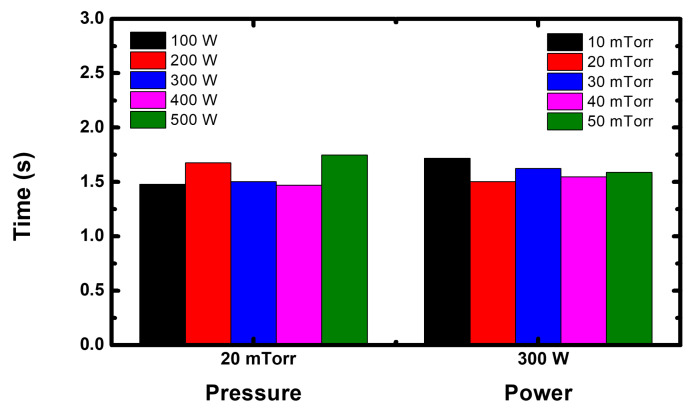
Calculation time for one IED with different pressure and RF power levels.

Figure 8 shows the calculation time of one IED as a function of pressure and power. This monitoring system includes the methods presented in the Section 3.3, and the vectorization of the code and loading method from the database of electron sheath edge motion, taking a calculation time of under 2 s overall RF power and pressure.

Unfortunately, it was impossible to compare the IEDs of both diagnostics adjacent to the powered electrode, and the IEDs under the condition that there was a dramatic change in sheath voltage waveforms because the commercial QMS could measure the IED adjacent to the grounded electrode due to the grounded sampling probe of the QMS, while our system had no constraint, whether powered or grounded. It is necessary to investigate further whether the IED was measured even under various changes in plasma conditions. As shown in Figure 6 and Figure 7, the IEDs calculated with our system were higher than those of the commercial QMS under all conditions. In order to find out the cause of this difference, it was necessary to measure the voltage waveform at the electrode and compare the difference with the voltage waveform at the point outside the cell where the probes were located. However, it was difficult to accurately measure the voltage waveform at the powered electrode due to plasma perturbation. Accordingly, although this difference was not clearly identified, it may have been caused by a stray impedance between the electrode and location of the probe [34]. Additionally, in actual plasma processing, fluorocarbon gas can produce numerous ion and neutral species in plasma, so improving this monitoring system that works even in this type of gas is our next work. Nevertheless, the results of this section show that this monitoring system is capable of noninvasively real-time monitoring the IED.

## 5. Conclusions

The invasive diagnostics of IEDs adjacent to a grounded or powered electrode during plasma processing are difficult for several reasons. Therefore, numerous studies have been conducted for noninvasive and real-time monitoring systems. Nevertheless, there are imperfect factors such as collision or noninvasive measurements.

In this paper, a noninvasive and real-time IED monitoring system was proposed and validated in an asymmetric RF CCP discharge in Ar plasma. Results show that changes in the IED measured via our system according to the input RF power and pressure variation were in good agreement with the results from a commercial QMS. This proposed monitoring system is capable of the noninvasive and real-time monitoring of IEDs in actual processing.

## Data Availability

The data presented in this study are available on request from the corresponding author.

## References

[1] Adamovich I. (2017). The 2017 Plasma Roadmap: Low temperature plasma science and technology. ESC J. Phys. D Appl. Phys..

[2] Oehrlein G.S., Metzler D., Li C. (2015). Atomic Etching at the Tipping Point: An Overview. ESC J. Solid State Sci. Technol..

[3] Kaler S.S., Lou Q., Donnelly V.M., Economou D.J. (2017). Atomic layer etching of silicon dioxide using alternating C_4_F_8_ and energetic Ar^+^ plasma beams. J. Phys. D Appl. Phys..

[4] Lee Y.S., Kim S.J., Lee J.J., Cho C.H., Seong I.H., You S.J. (2022). Purgeless atomic layer etching of SiO_2_. J. Phys. D Appl. Phys..

[5] Suto S., Hayasaka N., Okano H. (1989). Highly Selective Etching of Si_3_N_4_ to SiO_2_ Employing Fluorine and Chlorine Atoms Generated by Microwave Discharge. J. Electrochem. Soc..

[6] Hayashi H., Kurihara K., Sekine M. (1996). Characterization of Highly Selective SiO_2_/Si_3_N_4_ Etching of high-Aspect-Ratio Holes. Jpn. J. Appl. Phys..

[7] Kasternmeier B.E.E., Matsuo P.H., Oehrlein G.S. (1999). Highly selective etching of silicon nitride over silicon and silicon dioxide. J. Vac. Sci. Techno. A.

[8] Seman M., Wolden C.A. (2003). Investigation of the role of plasma conditions on the deposition rate and electrochromic performance of tungsten oxide thin films. J. Vac. Sci. Technol. A.

[9] Radjenovic B.M., Radmilovic-Radjenovic M.D., Petrovicm Z.L. (2008). Dynamics of the Profile Charging During SiO_2_ Etching in Plasma for High Aspect Ratio Trenches. IEEE Trans Plasma Sci..

[10] Brichon P., Pujo E.D., Mourey O., Joubert O. (2015). Key plasma parameters for nanometric precision etching of Si films in chlorine discharge. J. Appl. Phys..

[11] Gopikishan S., Banerjee L., Bigkem K.A., Das A.K., Pathak A.P., Mahapatra S.K. (2016). Paschen curve approach to investigate electron density and deposition rate of Cu in magnetron sputtering system. Radiat. Eff. Deffects Solids.

[12] Cho C.H., You K.H., Kim S.J., Lee Y.S., Lee J.J., You S.J. (2021). Characterization of SiO_2_ Etching Profiles in Pulse-Modulated Capacitively Coupled Plasmas. Materials.

[13] Kanarik K.J., Tan S., Gottscho R.A. (2018). Atomic layer etching: Rethinking the art of etch. J. Phys. Chem. Lett..

[14] Faraz T., Arts K., Karwal S., Knoops H.C.M., Kessels W.M.M. (2019). Energetic ions during plasma-enhanced atomic layer deposition and their role in tailoring material properties. Plasma Source Sci. Technol..

[15] Chang W.S., Yook Y.G., You H.S., Park J.H., Kwon D.C., Song M.Y., Yoon J.S., Kim D.W., You S.J., Yu D.H. (2020). A unified semi-global surface reaction model of polymer deposition and SiO_2_ etching in fluorocarbon plasma. Appl. Surf. Sci..

[16] Seong I.H., Lee J.J., Cho C.H., Lee Y.S., Kim S.J., You S.J. (2021). Characterization of SiO_2_ over poly-*Si* mask etching in and Ar/C_4_F_8_ capacitively coupled plasma. Appl. Sci. Converg. Technol..

[17] Chung Y.A., Lung C.Y., Chiu Y.C., Lee H.J., Lian N.T., Yang T., Chen K.C., Lu C.Y. Study of Plasma Arcing Mechanism in High Aspect Ratio Slit Trench Etching. Proceedings of the 2019 30th Annual SEMI Advanced Semiconductor Manufacturing Conference (ASMC).

[18] Carter D., Walde H., Nauman K. (2012). Managing arcs in large area sputtering applications. Thin Solid Film.

[19] Lee H.J., Seo D.S., May G.S., Hong S.J. (2013). Use of In-Situ Optical Emission Spectroscopy for Leak Fault Detection and Classification in Plasma Etching. J. Semicond. Technol. Sci..

[20] Marchack N., Buzi L., Farmer D.B., Miyazoe H., Papalia J.M., Yan H., Totir G., Engelmann S.U. (2021). Plasma prcessing for advanced microelectronics beyond CMOS. J. Appl. Phys..

[21] Simpson J.A. (1961). Design of Retarding Field Energy Analyzers. Rev. Sci. Instrum..

[22] Gahan D., Dolinaj B., Hopkins M.B. (2008). Retarding field analyzer for ion energy distribution measurements at a radio-frequency biased electrode. Rev. Sci. Instrum..

[23] Kreul S.G., Hübner S., Schneider S., Ellerweg D., Keudell A.V., Matejčík S., Benedikt J. (2015). Mass spectrometry of atmospheric pressure plasmas. Plasma Sources Sci. Technol..

[24] Benedikt J., Hecimovic A., Ellerweg D., Keudell A.V. (2012). Quadrupole mass spectrometry of reactive plasmas. J. Phys. D Appl. Phys.

[25] Singh H., Coburn J.W., Graves D.B. (2000). Appearance Potential Mass Spectrometry: Discrimination of Dissociative Ionization Products. J. Vac. Sci. Technol. A Vacuum Surfaces Film..

[26] Bohlmark J., Lattemann M., Gudmundsson J.T., Ehiasarian A.P., Gonzalvo Y.A., Brenning N., Helmersson U. (2006). The ion energy distributions and ion flux composition from a high power impulse magnetron sputtering discharge. Thin Solid Films.

[27] Ghidini R., Groothuis C.H.J.M., Sorokin M., Kroesen G.M.W., Stoffels W.W. (2004). Electrical and optical characterization of particle formation in an argon-silane capacitively coupled radio-frequency discharge. Plasma Sources Sci. Technol..

[28] Schauer J.C., Hong S., Winter J. (2004). Electrical measurements in dusty plasmas as a detection method for the early phase of particle formation. Plasma Sources Sci. Technol..

[29] Hong S., Berndt J., Winter J. (2002). Growth precursors and dynamics of dust particle formation in the Ar/CH_4 and Ar/C_2H_2 plasmas. Plasma Sources Sci. Technol..

[30] Shen Z., Kortshagen U. (2002). Experimential study of the influence of nanoparticle generation on the electrical characteristics argon-silane capacitive radio-frequency plasmas. J. Vac. Sci. Technol. A.

[31] Boufendi L., Gaudin J., Huet S., Viera G., Dudemaine M. (2001). Detection of particles of less than 5 nm in diameter formed in an argon-silane capacitively coupled radio-frequency discharge. Appl. Phys. Lett..

[32] Sezemsky P., Stranak V., Kratochvil J., Cada M., Hippler R., Hrabovsky M., Hubicka Z. (2019). Modified high frequency probe approach for diagnostics of highly reactive plasma. Plasma Sources Sci. Technol..

[33] Dewan N.A. (2001). Analysis and Modelling of the Impact of Plasma RF Harmonics in Semiconductor Plasma Processing. Ph.D. Thesis.

[34] Sobolewski M.A. (1992). Electrical characterization of radio-frequency discharges in the Gaseous Electronics Conference Reference Cell. J. Vac. Sci. Technol. A.

[35] Sobolewski M.A. (2001). Measuring the ion current in high-density plasmas using radio-frequency current and voltage measurements. J. Appl. Phys..

[36] Sobolewski M.A. (2004). Monitoring sheath voltages and ion energies in high-density plasmas using noninvasive radio-frequency current and voltage measurements. J. Appl. Phys..

[37] Sobolewski M.A. (2006). Real-time, noninvasive monitoring of ion energy and ion current at a wafer surface during plasma etching. J. Vac. Sci. Technol. A.

[38] Sobolewski M.A. (2005). Noninvasive monitoring of ion energy drift in an inductively coupled plasma reactor. J. Appl. Phys..

[39] Bogdanova M.A., Lopaev D., Zyryanov S.M., Rakhimov A.T. (2016). “Virtual IED sensor” at an rf-biased electrode in low-pressure plasma. Phys. Plasmas.

[40] Bogdanova M.A., Lopaev D., Rakhimov A.T., Zotovich A., Zyryanov S.M. (2021). ‘Virtual IED sensor’ for df rf CCP discharges. Plasma Sources Sci. Technol..

[41] Bogdanova M.A., Lopaev D., Zyryanov S.M., Voloshin D., Rakhimov A.T. (2019). Ion composition of rf CCP in Ar/H_2_ mixtures. Plasma Sources Sci. Technol..

[42] Metze A., Erine D.W., Oskam H.J. (1986). Application of the physics of plasma sheaths to the modeling of rf plasma reactors. J. Appl. Phys..

[43] Bruce R.H. (1981). Ion response to plasma excitation frequency. J. Appl. Phys..

[44] Vahedi V., Surendra M. (1995). A Monte Carlo collision model for the particle-in-cell method: Applications to argon and oxygen discharges. Comput. Phys. Commun..

[45] Lieberman M.A., Lichtenberg A.J. (2005). Principles of Plasma Discharges and Materials Processing.

[46] Bohm D. (1949). The Characteristics of Electrical Discharges in Magnetic Fields.

[47] Dai Z.L., Wang Y.N. (2004). Simulations of ion transport in a collisional radio-frequency plasma sheath. Phys. Rev. E.

[48] Skullerud H.R. (1968). The stochastic computer simulation of ion motion in a gas subjected to a constant electric field. J. Phys. D Appl. Phys..

[49] Köhler K., Coburn J.W., Horne D.E., Kay E., Keller J.H. (1985). Plasma potentials of 13.56MHz rf argon glow discharges in a planar system. J. Appl. Phys..

[50] Edelberg E.A., Perry A., Benjamin N., Aydil E.S. (1999). Energy distribution of ions bombarding biased electrodes in high density plasma reactors. J. Vac. Sci. Technol. A..

[51] Rusu I.A., Popa G., Sullivan J.L. (2002). Electron plasma parameters and ion energy measurement at the grounded electrode in an rf discharge. J. Phys. D Appl. Phys..

[52] Chen F.F. (2018). Introduction to Plasma Physics and Controlled Fusion.

[53] Chabert P., Braithwaite N. (2011). Physics of Radio-Frequency Plasmas.

